# How does age affect personal and social reactions to COVID-19: Results from the national Understanding America Study

**DOI:** 10.1371/journal.pone.0241950

**Published:** 2020-11-10

**Authors:** Jung Ki Kim, Eileen M. Crimmins

**Affiliations:** Davis School of Gerontology, University of Southern California, Los Angeles, California, United States of America; University of California-Irvine, UNITED STATES

## Abstract

The COVID-19 pandemic has had tremendous impact on Americans’ lives including their personal and social behaviors. While people of all ages are affected in some way by the pandemic, older persons have been far more likely to suffer the most severe health consequences. For this reason, how people have responded to mitigating behaviors to COVID-19 may differ by age. Using a nationally representative sample from the longitudinal data of the Understanding America Study (UAS), we examined differentials in behavioral responses to COVID-19 by age and how they changed over the first three months of the pandemic. Behavioral responses and changes in behavior over time differed by age, type of behaviors and time reference. At the beginning of the pandemic (March, 2020), older and younger people were similar in their likelihood of engaging in preventive personal behaviors when controlling for other influences. As the pandemic progressed, however, older people adopted mitigating personal behavioral changes more than younger people, such that about 1–2 months after the pandemic started, older people were more likely to comply with suggested behaviors and regulations including practicing better hygiene, quarantining, and social distancing. One month into the pandemic, older people were less likely than younger people to engage in two of four risky behaviors. The change in risky behavior over time did not differ by age; but both younger and older people were more likely to engage in risky behaviors after two months. Being female, a member of a racial/ethnic minority group, higher socioeconomic status, having more COVID-19 cases in one’s state of residence, a higher perceived risk for infection and dying, and a more left-leaning political orientation were related to adopting more pandemic mitigating behaviors.

## Introduction

As the COVID-19 pandemic has spread across America, dramatic change has occurred in many aspects of personal and social life. Quarantine and social distancing have been mandated in many states to prevent and reduce further spread of COVID-19. Across the country, guidelines for individual behavior have been publicized. While the COVID-19 pandemic appears to have peaked in some places, in others it continues unabated or is still increasing. We may reach a point where new cases and deaths decline, or another peak may occur when the flu season and COVID-19 pandemic occur simultaneously. Public health practices including household-based quarantine, avoiding social interactions at close distance, avoiding crowds, washing hands, wearing a mask and identification and isolation of cases are the recommended approaches to reducing the number of cases infected by this highly contagious virus [1], and controlling the spread of the virus [2]. Previous studies have shown that older people are more likely to practice healthy behaviors both in general [3,4] and in epidemic situations [5], particularly women [6]. Older people may be more conscientious and more cautious about recommended health practices because the consequences of unhealthy practices may be more severe and fatal [4]; while younger people may not suffer fatal consequences of unhealthy choices [7].

The risk of developing severe COVID-19 complications or dying increases dramatically with age [8,9]. About 79% of COVID-19 deaths reported by August 29, 2020 occurred at ages 65+, and the population fatality rate (the risk of death from COVID-19 among the general population) dramatically increased by age so the rate was 0.82% for ages 85+, 0.29% for ages 75–84, 0.12% for ages 65–74, 0.05% for ages 55–64, 0.02% for ages 45–54, compared to 0.003% for ages under 45 [10]. Fatality rates are always the highest among older people, across countries with varying levels of COVID-19 mortality and infection [11,12]. This appears to result from the fact that higher age is linked to more underlying health conditions and weaker immune systems [8,9,12,13].

Because of increased risk from COVID-19, older people should have responded to the COVID-19 pandemic by engaging in more preventive behaviors and avoiding more risky behaviors. Some earlier research relevant to epidemics has suggested that being older is related to greater compliance and participation in practicing precautionary behaviors [14,15]; however, not all results are consistent in finding a strong age difference [16]. In addition, how soon people adopt healthy reactions may vary by age. Previous findings show that during the outbreak of the 2009 influenza A (H1N1) pandemic, older age was not related to taking preventive measures such as practicing better hygiene, avoiding persons with influenza-like symptoms and avoiding crowded places, but it was related to stronger intentions to comply with government-advised preventive measures in the future [17]. Once the spread of infection increases, people are more aware of the pandemic, and after governmental advice on practicing preventive behaviors is phased in [17], older people may take more precautions.

Behavioral change may differ by age, but it could also change over time. While we expect that as people become more aware of the risks, they will adopt the recommended behavioral modifications; however, it is possible that people may lose interest in continuing to practice recommended behaviors after an initial period [18]. In addition, this time dynamic may depend on the type of behaviors investigated. People may continue to abide by personal preventive behaviors such as wearing masks and personal hygiene; people may differ in their willingness to forgo social interactions for a longer term. People may also grow used to the pandemic and relax their behaviors over time. It is not clear how this time dynamic will be related to age.

In this paper, we use data collected between March 10 and March 31, 2020 (wave 1), between April 1 and April 28, 2020 (wave 2), and between April 29 and May 26, 2020 (wave 4), from a nationally representative sample of American adults to examine how adults of different ages have responded to public health recommendations for behavioral changes to control the spread of COVID-19 and how the response of different age groups changed over time. We compare the fourth wave of the data with the first two waves from the survey to investigate whether behavioral change was greater among older people as the pandemic progressed.

While our focus is on the relationship of age with adopting behavioral responses as the increased level of underlying health conditions among older people is linked to higher risk of severe disease consequences [8,9,12], we are also interested in how other individual characteristics are related to the adoption of COVID-19 mitigating behaviors. Some of these factors may also be related to age and thus are necessary as controls, and others may be related to actual or perceived risk or ability to comply with recommendations. Males may be less likely to take preventative actions and more likely to engage in risky behaviors [6]. Those with lower income may be disadvantaged in adopting preventive pandemic reactions and take more risky behaviors out of necessity due to their lower socioeconomic status and associated occupational statuses and need to work and to use public transportation [19]. This may be particularly true for members of racial and ethnic minority groups. However, the significant spread of information on the higher rate of COVID-19 cases and disproportionate death toll among Black and Hispanic Americans [12] may have alerted them to a greater need for precautionary behaviors. Living with someone else may make quarantining and social distancing easier to adapt to than for those living alone. Being an essential employee may require a person to engage in more risky health behaviors in order to maintain financial well-being. As the pandemic varied markedly in its course across geographic regions, those who reside in states where the COVID-19 outbreak is more widespread and where there are more cases, may be more likely to take actions to maintain their health. Those who trust the current government and media that portrays the COVID-19 crisis with skepticism and downplays the danger of the virus are less likely to take preventive behaviors and more likely to take risky behaviors compared to those who trust media sources that portray the pandemic as risky and endorse public health recommendations [20–24]. Those who perceive a higher personal probability of getting the infection and dying are more likely to respond to behavioral modifications than those whose assessment of their risk for infection and death is low. All of these factors may influence how people practice preventative and risky behaviors in response to COVID-19, and influence differential response by age.

## Materials and methods

### Participants and design

We used the Understanding Coronavirus in America Tracking Survey from the national Understanding America Study (UAS). This is an ongoing COVID-19 tracking survey which has been incorporated into the UAS probability-based internet panel data. The UAS is an ongoing nationally representative internet panel of respondents aged 18 and over, supported by the Social Security Administration (SSA) and the National Institute on Aging, and administered by the Center for Economic and Social Research (CESR) at the University of Southern California (USC) [24]. Participants complete surveys on their own time with their internet devices such as a computer, tablet, or smart phone and they are provided with a tablet and internet access if needed [25,26]. Participants in the survey were UAS panel members who are randomly selected based on U.S. addresses in the Computerized Delivery Sequence (CDS) file which covers almost all or 100% of U.S. households [25]. The UAS oversamples Native Americans and residents of Los Angeles County and California; however, the data are weighted to produce a national sample. In the Understanding Coronavirus in America survey, the panel members are invited to participate in a survey over a 14-day period and they have 2 weeks to complete the survey; however, they receive an extra monetary incentive if they complete the survey on the day invited. The UAS COVID-19 data are secondary data to us. We did not obtain the informed consent from the participants. The survey participants log into the UAS website and provide consent before they take the first survey [25].

Beginning with the first wave collected March 10 through March 31, the Understanding Coronavirus in America tracking survey released 4 waves as by May 27, 2020. We used the three waves (wave 1 (3/10-3/31); wave 2 (4/01-4/28); wave 4 (4/29-5/26)) for analysis of differentials by age and other factors and of change in personal and social responses over the first 2 month period after the pandemic started. Among 8,500 eligible panel members, 6,932 (82%) in wave 1, 5,478 (64%) in wave 2, and 6,403 (75%) in wave 4 participated in the survey. Our analysis begins with 5,128 participants who responded to all three waves. The final analytic samples vary depending on the behavioral outcome and time-varying covariates in each model, ranging from 4,690 to 4,825 for comparison between wave 1, wave 2 and wave 4, and from 4,759 to 4,820 for comparison between wave 2 and wave 4. Because the sample is chosen with varying probabilities [25,26], we used the final post-stratification sample weight to align the sample with the whole U.S. adult population (ages 18+) along a set of sociodemographic dimensions such as age, gender, race/ethnicity, education and location [27]. In this sample, very few respondents had been diagnosed with coronavirus by a healthcare professional; only 0.04% were diagnosed at wave 1; 0.58% by wave 2; and 0.78% by wave 4.

### Measures

#### Personal and social behaviors in response to COVID-19

We examined 10 personal and social behaviors reported as taken in the last seven days in response to the COVID-19 pandemic. The behaviors were divided into six preventive personal activities which people are expected and encouraged to do in order to prevent the spread of the virus infection and four risky social behaviors that could increase the likelihood of infection. Each behavior was coded as yes (1) or no (0).

The preventive personal behaviors include: (1) wore a mask or other face covering, (2) washed hands with soap or used hand sanitizers several times a day, (3) canceled or postponed personal or social activities, (4) avoided contact with people who could be high-risk, (5) avoided public spaces, gatherings, or crowds, and (6) avoided eating at restaurants. These six behaviors were available at waves 1, 2 and 4.

Risky social behaviors include (1) went to a friend, neighbor, or relative’s residence (not their own), (2) had close contact (within 6 feet) with people who do not live with them, (3) attended a gathering with more than 10 people, such as a reunion, wedding, funeral, birthday party, concert, or religious service, and (4) had visitors such as friends, neighbors or relatives at their residence. These were not asked at wave 1, but at waves 2 and 4.

#### Sociodemographic measures

The respondents ranged in age from 18 to 101: categorized into 4 groups, 18–34, 35–54, 55–64, and 65+. Education was divided into four categories (less than high school (0–11 years), high school graduate (12 years), some college (13–15 years), and college graduate (16+ years)). Race/ethnicity was divided into non-Hispanic white, non-Hispanic Black, Hispanic, non-Hispanic Asian and “other” which includes mixed race/ethnicity. Living arrangements were categorized into living alone vs living with someone else. Health status was measured by the number of underlying chronic conditions including diabetes, cancer, heart disease, high blood pressure, asthma, chronic lung disease, kidney disease, autoimmune disorder, mental health condition and obesity. This health measure is available only at wave 4, so this is a time-invariant variable. Employment status was dichotomized into whether a respondent currently had a job or not. Poverty was defined as a household income at or lower than the 2020 U.S. federal poverty level issued by U.S. Department of Health and Human Services [28], calculated by dividing household income by the number of household members in each wave. Each state’s COVID-19 case numbers were used to indicate geographic variation in exposure to COVID-19 [29]. We used the number of COVID-19 cases, 1–3 weeks prior to the survey date to reflect the possible lag between the changing numbers of cases and the change in people’s behaviors. For each wave, we divided the respondents into two groups, those who answered during the first half of the survey period, and those who answered in the last half. Then, we assigned the number of cases in each state from one week prior to the survey start date. Those who started their survey between 3/10/20 and 3/20/20 were assigned state case numbers from March 3; those who started the survey between 3/21 and 3/31 were assigned the number of cases from March 14; state case numbers from 3/25 for those whose survey started between 4/1 and 4/14; 4/8 for surveys started between 4/15 and 4/28; 4/22 for surveys started between 4/29 and 5/12; and 5/6 for surveys started between 5/13 and 5/26. Thus, the number of state COVID-19 infection cases was time varying. To indicate political leaning, we used people’s rating of their trust in media sources, particularly Fox News and CNN. We categorized media trust into three groups: trusting Fox News more, trusting CNN more, and equal or no trust in Fox News and CNN [24]. Finally, people provided their assessment of their personal likelihood of becoming infected with COVID-19 and of dying from the condition in the next 3 months. Responses scaled from 0 to 100, with 100 indicating the highest probability. While it would be ideal to specify residential setting by adding urban and rural distinctions and by distinguishing living in an individual household or in a residential living facility, the UAS COVID-19 data do not provide this information.

### Analysis

First, the percent of people who practiced each COVID-19 related behavior or activity was examined by age at three time points (wave 1, wave 2, and wave 4) for the first six behaviors and at two time points (wave 2 and wave 4) for the last four behaviors. Then, each behavior was regressed using logistic regression models on all other variables potentially related to people’s responses to examine whether age is still related to responses with these variables controlled. We examined how different age groups changed COVID-19 related behaviors over time by including time (wave) and time and age interactions in the models. Since multiple respondents could come from the same household, we adjusted for household clustering as well as individual clustering as we included responses from individuals at multiple waves. We employed a multilevel (three level) clustering adjustment using both household and respondent identifiers. The sample Stata code we have used is as follows:

*melogit beh_facemask wave i.agecat i.agecat##wave female livingalone i.race i.education numdisease currentjob poverty state i.mediatrust prisk_die prisk_infection state2-state51 [pweight = final_weight]*, || *uashhid*: || *uasid*:, *or*

## Results

Table 1 provided descriptive information on the sample and the variables used in this study. In wave 4, about 24.3% (N = 1,244) of our sample was age 65 and over; 18.1% (N = 928) was age 18–34, 36.8% (N = 1,887) was 35–54, and 20.8% (N = 1,069) was age 55–64. The majority of the oldest age group was non-Hispanic white (82.3%) while the percent in this category was lower at younger ages: the percentages for ages 18–34, 35–54 and 55–64 were 57.4%, 58.4% and 67.7%, respectively. A number of additional characteristics differed across age groups that could affect the reported behavioral responses by age. The percent living alone was almost double for those at older ages relative to younger ages. The number of chronic diseases was also greater for the oldest age group. While more than half were currently working at younger ages, only 14.6% of the oldest group were working at the time of survey. About 19.4% trusted Fox News more than CNN and 28.1% trusted CNN more. The percent trusting Fox News more increased with age such that the percent trusting Fox News more than CNN among those aged 65+ (30.2%) was three times greater than that among those aged 18 to 34 (10.1%). The percent who did not trust either media source or equally trust the two declined with age. The average perceived risk of dying was double among the oldest age group compared to the youngest age group (31.4 vs 15.8). The level of perceived risk for dying or infection was highest at wave 2 and the lowest at wave 1. The average number of COVID-19 cases in the state of residence increased dramatically from March to May, but it was similar across age groups at wave 4. While most characteristics of the respondents were quite similar across waves, the percent having a job dropped by 10% from wave 1 to wave 2/wave 4.

**Table 1:** Description of the UAS national sample: Waves 1, 2 and 4.

	Wave 1 (03/10/20-03/31/20)	Wave 2 (04/01/20-04/28/20)	Wave 4 (04/29/20-05/26/20)				
Age (years)	Total	Total	Total	18–34	35–54	55–64	65+

% Female	49.7%	51.9%	50.7%	60.7	50.9	49.3	41.0
% Living alone	17.3%	16.9%	16.9%	12.5	12.1	23.1	23.9
% Race/ethnicity							
White	66.0%	63.6%	65.1%	57.4	58.4	67.7	82.3
Black	11.1%	11.6%	11.5%	12.2	13.3	11.4	7.7
Hispanic	14.2%	16.2%	15.1%	20.3	18.8	14.3	4.1
Asian	5.3%	5.2%	5.0%	6.5	6.0	3.3	3.3
Other	3.4%	3.4%	3.3%	3.7	3.6	3.4	2.7
% Education (years)							
0–11	36.8%	38.0%	37.4%	35.0	35.6	41.0	39.6
12	27.9%	27.4%	27.6%	31.9	25.0	31.1	24.8
13–15	19.6%	19.2%	19.4%	20.6	20.7	17.2	17.9
16+	15.7%	15.4%	15.6%	12.5	18.7	10.7	17.8
Mean (SD) number of chronic conditions at wave 4	1.12 (1.31)	1.11 (1.30)	1.11 (1.30)	0.63 (0.99)	0.89 (1.20)	1.36 (1.29)	1.76 (1.38)
% Currently working	59.5%	49.5%	49.1%	55.6	66.8	46.8	14.6
% In Poverty	13.8%	14.6%	14.6%	22.9	15.6	9.8	8.6
Mean (SD) COVID-19 cases per 1,000 in state of residence	0.01 (0.05)	3.06 (8.36)	31.76 (54.57)	33.21 (62.36)	28.95 (50.95)	35.36 (57.91)	31.87 (50.37)
% Political inclination at wave 1							
Trust Fox News more	19.7%	19.1%	19.4%	10.1	16.2	24.2	30.2
Trust Fox News/CNN equally or no Trust	52.0%	53.0%	52.5%	64.0	56.2	50.7	36.1
Trust CNN more	28.3%	27.9%	28.1%	25.9	27.6	25.1	33.8
Mean (SD) perceived risk for infection	20.5 (22.4)	27.7 (23.4)	23.8 (21.5)	24.3 (24.2)	25.3 (22.4)	22.2 (19.6)	22.2 (19.2)
Mean (SD) Perceived risk for dying	15.3 (22.6)	25.1 (26.5)	21.2 (25.3)	15.8 (22.9)	17.6 (23.3)	22.5 (23.7)	31.4 (28.3)
N	5,128	5,128	5,128	928	1,887	1,069	1,244

**Fig 1:**
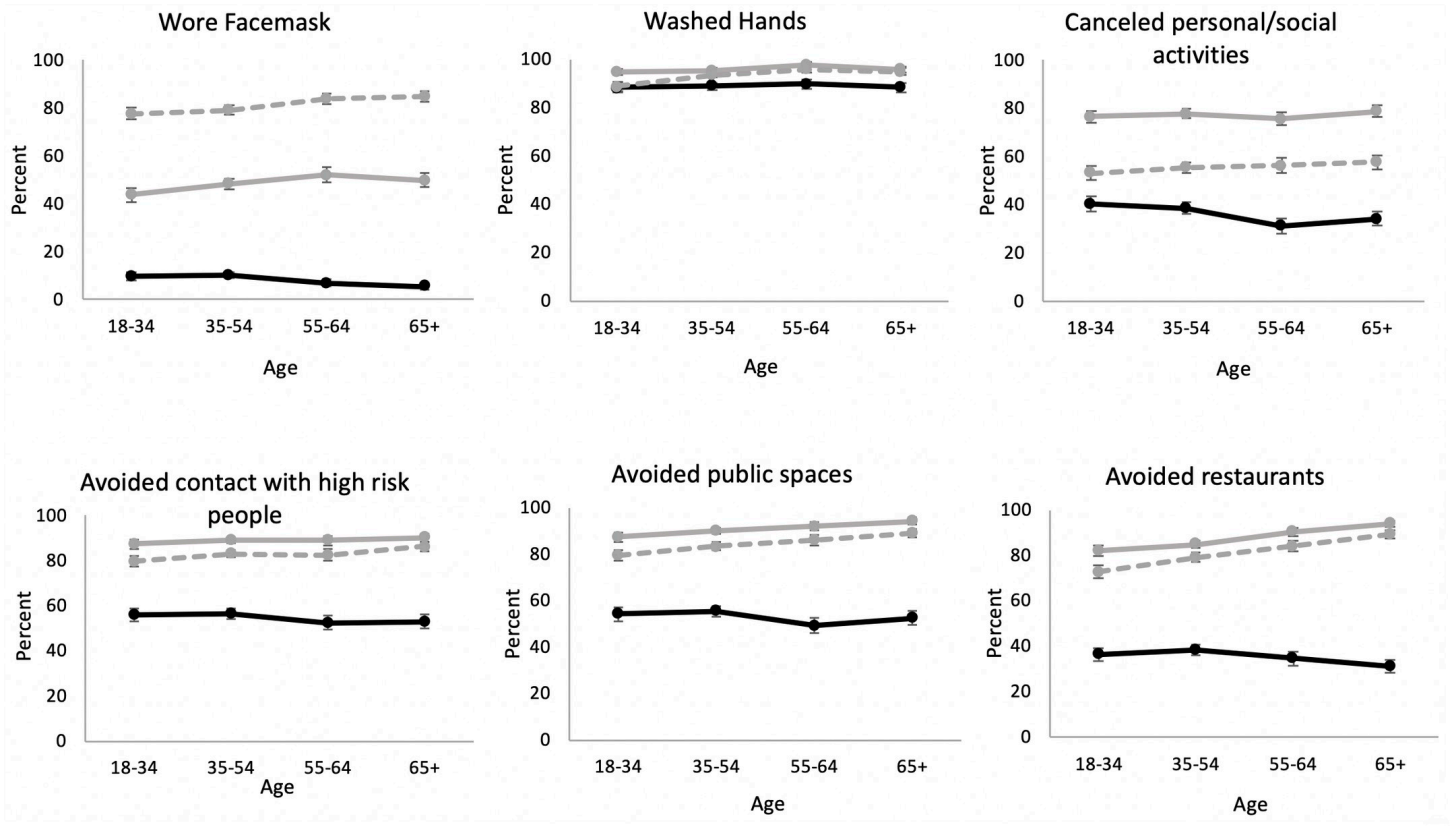
Percent of people who performed preventive personal behaviors in response to COVID-19 by age: Wave 1 (03/10/20-03/31/20), wave 2 (04/01/20-04/28/20) and wave 4 (04/29/20-05/26/20).

Comparison of the percent taking each preventive behavior by age in March (3/10 to 3/31), one month later in April (4/1/20-4/28/20) and two months later in May (4/29 to 5/26) indicates that reactions to COVID-19 changed over time as this pandemic has progressed and these changes differed by age (Fig 1). At the beginning of the quarantine period, older people were no more likely than younger people to practice preventive behaviors in response to the pandemic. In March, older people were less likely than younger people to wear a facemask, wash hands, cancel activities, and avoid high-risk people, public places and restaurants, but by May, older people were more likely to implement such behaviors. Change between March and May was smaller for the youngest age group and greatest for the oldest age group. Except for wearing a mask, people adopted preventive activities in the first month but then reduced the modification of their behaviors some after April so that the percent taking these preventive behaviors was lower in May than April. That is, the behavioral changes were made quickly in the initial month after the beginning of the pandemic. However, the use of facemasks continued to increase over time such that the percent in May was about double that in April.

**Fig 2:**
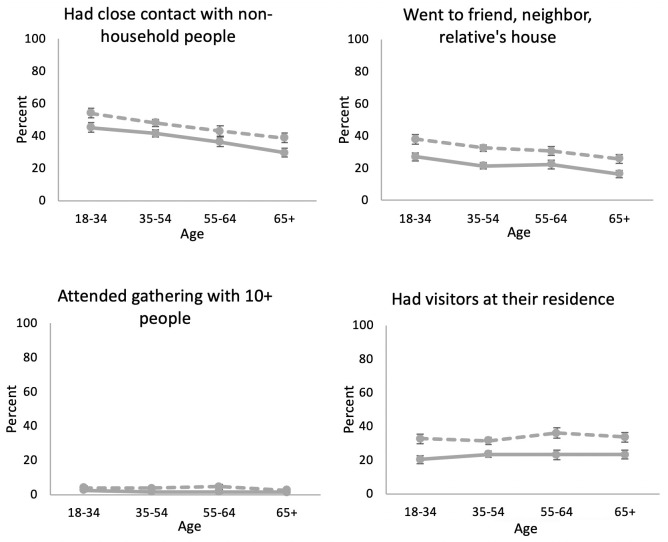
Percent of people who performed risky social behaviors in response to COVID-19 by age: Wave 2 (04/01/20-04/28/20) and wave 4 (04/29/20-05/26/20).

Because the questions about some behaviors began in wave 2, we compared the percent who had close contact with non-household people, who went to other people’s houses, who attended gatherings with more than 10 people, and who had visitors at waves 2 and 4, approximately a month apart, between April and May. The percent engaging in most risky behaviors was lower at older ages (Fig 2). At wave 4, about 54.0% of those aged 18–34 had close contact with non-household people while the percent was much lower for the oldest age group (38.7%). Similarly, the percent going to a friend, neighbor or relative’s residence was lower at older ages. While very low overall, those aged 65 and over were less likely to attend a gathering with more than 10 people than those aged 18–34 (2.6% vs 3.8%). Only the percent having visitors at their residence did not differ across age (about 33% for both the youngest and the oldest age group). While older people were less likely to practice each risky behavior both at waves 2 and 4, people engaged in more risky behaviors in all age groups at the later wave.

Next, we examined how age was related to preventive personal responses to COVID-19 with other potential influences controlled in multivariate analyses and how the relationship between age and behavioral responses changed over time, that is at waves 2 and 4 compared to wave 1. With controls for gender, living arrangements, education, race/ethnicity, health status, employment status, poverty, the COVID-19 number of cases in the state of residence, political orientation, and self-assessed risk for infection and dying, at wave 1, age was not related to the likelihood of engaging in healthy behaviors in response to COVID-19 (Table 2 and S1 Table). At wave 2, all behaviors were more likely to have been adopted by panel members aged 18 to 34; most behaviors were still more likely in this age group by wave 4 (except hand washing), although the adoption was becoming less, except for mask wearing. People aged 35 to 54 did not change behavior much over time. In general people over the age of 55 were more likely to adopt virus mitigating behaviors than those aged 18–34 by wave 4. By wave 4, those 65 and older had a greater likelihood of engaging in three out of the six mitigating behaviors. At wave 4, the oldest people (65+) were 290% to 570% more likely to adopt preventive personal behaviors relative to those ages 18 to 34. At wave 4, the odds of wearing a facemask were 2.9 times (= 0.73*3.94, 95% CI: 1.18–6.95, p = .004) greater for the oldest group than for the youngest group. Those in the oldest ages were 5.7 times more likely to wash hands (= 1.45*3.90, 95% CI: 1.56–20.43, p = < .000), 3.1 times more likely to avoid public places (= 1.06*2.90, 95% CI: 1.24–7.54, p = .002) and 5.5 times more likely to avoid eating at a restaurant (= 0.80*6.85, 95% CI: 2.40–12.60, p = < .001) than for the youngest group. Those aged 55–64 were more likely to engage in 4 of the 6 behaviors (OR = 2.7 (0.73*3.64), 95% CI: 1.09–6.48, p = .004 for wearing a mask; OR = 6.0 (1.27*4.73), 95% CI: 1.70–21.30, p < .001 for washing hands; OR = 2.8 (0.96*2.96), 95% CI: 1.21–6.69, p = .002 for avoiding public places; OR = 3.1 (1.02*3.01), 95% CI: 1.4806.40, p < .001 for avoiding restaurants); they did not differ from the youngest group in the likelihood of canceling activities and avoiding high-risk people. Some of these changes among people over 55 had occurred by wave 2: wearing masks, avoiding public places and avoiding restaurants. The association between age and these preventive behaviors was significantly changed over time. Older and younger people did not differ in the odds of taking these preventive behaviors in the beginning of the pandemic, but in the first months, older people quickly adopted more preventive personal behaviors relative to the youngest people. Based on the results from Table 2 where all background factors were controlled, we calculated and plotted the predicted probabilities (S1 Fig) to visually display the trend in behavioral modifications by age. The plots were quite similar to the descriptive figure (Fig 1).

**Table 2:** Odds ratios for age and other factors related to preventive personal behaviors: Wave 1 (03/10/20-03/31/20), wave 2 (04/01/20-04/28/20) and wave 4 (04/29/20-05/26/20).

	Wore mask		Washed hands		Canceled personal/social activities		Avoided high-risk people		Avoided public places		Avoided restaurants	
	OR	P	OR	p	OR	p	OR	p	OR	p	OR	p
Age (18–34 years as reference)												
35–54	0.98	0.946	0.98	0.950	0.97	0.859	1.04	0.813	1.16	0.405	1.18	0.385
55–64	0.73	0.344	1.27	0.449	0.73	0.103	0.98	0.917	0.96	0.850	1.02	0.917
65+	0.73	0.370	1.45	0.247	0.90	0.577	0.95	0.813	1.06	0.798	0.80	0.301
Wave (wave 1 as reference)												
Wave 2	28.33	< .001	4.14	< .001	10.57	< .001	15.37	< .001	19.25	< .001	29.77	< .001
Wave 4	410.96	< .001	1.03	0.929	2.29	< .001	6.42	< .001	7.68	< .001	12.35	< .001
Age*wave interaction												
Age 35–54*wave 2	1.50	0.244	1.29	0.588	1.13	0.613	1.02	0.951	1.17	0.616	1.18	0.590
Age 35–54*wave 4	1.21	0.646	2.74	0.009	1.14	0.541	1.22	0.466	1.34	0.292	1.44	0.165
Age 55–64*wave 2	3.16	0.002	2.66	0.064	1.56	0.105	1.23	0.519	3.00	0.002	3.19	< .001
Age 55–64*wave 4	3.64	0.002	4.73	0.001	2.08	0.002	1.44	0.229	2.96	< .001	3.01	< .001
Age 65+*wave 2	3.05	0.004	1.20	0.726	1.47	0.133	1.35	0.346	3.37	0.001	7.23	< .001
Age 65+*wave 4	3.94	0.002	3.90	0.002	1.66	0.028	1.98	0.021	2.90	0.001	6.85	< .001
Female	1.42	< .001	2.61	< .001	1.30	< .001	1.30	0.002	1.31	0.003	1.26	0.005
Living alone	0.91	0.451	0.65	0.018	0.81	0.029	0.89	0.308	0.77	0.037	0.87	0.239
Race/ethnicity (white as reference)												
Black	4.66	< .001	3.39	< .001	1.66	0.001	2.09	< .001	1.73	0.005	2.07	< .001
Hispanic	2.12	< .001	3.59	< .001	2.20	< .001	1.70	0.003	2.13	< .001	1.83	< .001
Asian	3.26	< .001	1.58	0.278	1.94	0.001	1.38	0.219	3.03	< .001	3.40	< .001
Other	1.73	0.011	1.54	0.194	1.12	0.525	1.39	0.176	1.09	0.725	1.15	0.561
Education (16+ years as reference)												
13–15 years	0.92	0.542	1.04	0.844	1.09	0.431	0.96	0.767	1.21	0.189	1.14	0.311
12 years	1.02	0.867	0.88	0.545	0.77	0.011	0.85	0.211	0.70	0.010	0.86	0.252
0–11 years	0.65	0.008	0.74	0.219	0.64	0.000	0.95	0.747	0.72	0.039	0.87	0.359
Number of chronic conditions	1.12	0.005	1.14	0.026	0.96	0.185	1.00	0.930	1.04	0.323	0.99	0.828
Currently having a job	1.50	0.001	1.51	0.022	0.93	0.436	0.80	0.044	0.76	0.018	0.73	0.005
In poverty	1.07	0.687	0.65	0.053	0.89	0.370	0.88	0.400	0.87	0.425	0.91	0.570
Number of COVID-19 cases in the state of residence (per 1,000)	1.01	0.004	1.00	0.397	1.00	0.836	1.00	0.677	1.00	0.477	1.00	0.054
Political inclination (equal trust or no trust as reference)												
Trust Fox news more	0.87	0.278	1.08	0.690	0.92	0.408	0.87	0.236	0.91	0.418	0.72	0.006
Trust CNN more	1.48	< .001	1.63	0.006	1.28	0.003	1.21	0.063	1.63	< .001	1.51	< .001
Perceived risk for infection	1.01	0.001	1.00	0.423	1.01	0.001	1.01	0.016	1.01	0.007	1.01	0.008
Perceived risk for dying	1.00	0.097	1.01	< .001	1.01	< .001	1.01	< .001	1.01	< .001	1.01	0.001
N	4,825		4,825		4,805		4,690		4,812		4,812	
Wald χ2	839.43		266.48		886.36		683.70		785.00		931.79	
Prob > χ2	0.0000		0.0000		0.0000		0.0000		0.0000		0.0000	
Log pseudolikelihood	-5544.50		-2803.69		-7711.72		-5971.95		-5581.58		-5979.43	

We also examined how taking risky social behaviors differed across age groups at waves 2 and 4. At wave 2, the oldest age group was less likely than the youngest age group to have close contact with non-household people (OR = 0.44, 95% CI: 0.28–0.69, p < .001) and go to other people’s residence (OR = 0.37, 95% CI: 0.22–0.63, p < .001) (Table 3 and S2 Table). At wave 4, the odds of having close contact with non-household people for the oldest age group were still 59% less than for the youngest age group (OR = 0.41 (0.44*0.94), 95% CI: 0.20–0.82, p = .002) and those of going to friend’s residence were 67% less than for the youngest age group (OR = 0.33 (0.37*0.89), 95% CI: 0.15–0.73, p = < .001). The odds of taking these risky behaviors for the oldest age group increased from wave 2 to wave 4 and the odds of taking risky behaviors for those aged 18–34 at wave 4 relative to wave 2 were also increased for three behaviors: having contact with non-household members, going to another residence, or having visitors. While both younger and older people took more risky behaviors by wave 4, the older group still engaged in fewer risky behaviors relative to the youngest. Using the results from Table 3, the predicted probabilities were plotted (S2 Fig), which again looked similar to what was plotted in Fig 2.

**Table 3:** Odds ratios for age and other factors related to risky social behaviors: Wave 2 (04/01/20-04/28/20) and wave 4 (04/29/20-05/26/20).

	Had close contact with non-household people		Went to friend, relative’s residence		Attended gathering of 10+ people		Had visitors in residence	
	OR	p	OR	p	OR	p	OR	p
Age (18–34 years as reference)								
35–54	0.74	0.126	0.58	0.019	0.68	0.488	1.19	0.487
55–64	0.56	0.010	0.66	0.114	0.53	0.320	1.12	0.669
65+	0.44	< .001	0.37	< .001	0.73	0.624	1.26	0.408
Wave (wave 2 as reference)								
Wave 4	1.50	< .001	1.80	< .001	1.47	0.174	1.88	< .001
Age*wave interaction								
Age 35–54*wave 4	0.78	0.319	0.96	0.883	1.29	0.720	0.64	0.140
Age 55–64*wave 4	0.77	0.327	0.76	0.370	3.08	0.141	0.91	0.769
Age 65+*wave 4	0.94	0.799	0.89	0.705	1.06	0.943	0.74	0.330
Female	0.80	0.021	0.93	0.539	0.92	0.674	0.96	0.724
Living alone	1.54	0.001	1.87	< .001	1.41	0.215	1.05	0.756
Race/ethnicity (white as reference)								
Black	0.99	0.962	0.82	0.420	1.23	0.620	0.87	0.557
Hispanic	1.10	0.630	0.72	0.154	1.58	0.200	0.99	0.955
Asian	0.71	0.221	0.68	0.222	1.22	0.759	0.51	0.046
Other	1.78	0.016	1.40	0.230	2.43	0.077	1.70	0.035
Education (16+ years as reference)								
13–15 years	1.28	0.098	1.09	0.610	0.65	0.226	0.99	0.944
12 years	1.97	< .001	1.27	0.169	0.71	0.325	1.44	0.039
0–11 years	1.49	0.021	1.16	0.456	0.57	0.123	1.54	0.028
Number of chronic conditions	1.01	0.811	0.97	0.486	0.91	0.316	1.09	0.067
Currently having a job	2.49	< .001	1.30	0.061	1.35	0.296	1.23	0.151
In poverty	0.69	0.027	0.81	0.297	1.44	0.291	1.01	0.944
Number of COVID-19 cases in the state of residence (per 1,000)	1.00	0.236	1.00	0.379	1.00	0.579	1.00	0.657
Political inclination (equal trust or no trust as reference)								
Trust Fox news more	1.11	0.419	1.19	0.249	1.49	0.145	1.43	0.020
Trust CNN more	0.79	0.045	0.62	< .001	0.46	0.008	0.62	0.001
Perceived risk for infection	0.99	< .001	1.00	0.113	0.99	0.150	1.00	0.075
Perceived risk for dying	1.01	0.006	1.00	0.772	1.00	0.873	1.00	0.813
N	4,759		4,820		4,819		4,815	
Wald χ2	298.04		265.40		105.48		217.25	
Prob > χ2	0.0000		0.0000		0.0000		0.0000	
Log pseudolikelihood	-5189.99		-4386.59		-915.73		-4451.71	

In addition to age, other factors were related to people’s behaviors. Females were more likely than males to take preventive responses while they did not differ in risky behaviors except having close contact with non-household people (OR = 0.80, 95% CI: 0.66–0.97, p = 0.021). Being Black or Hispanic was strongly related to a higher likelihood of engaging in healthy behaviors across different types of preventive behaviors, and other racial/ethnic minorities such as Asians and other groups were also more likely than whites to take most preventive behaviors. Black Americans were more likely than whites to wear a facemask (OR = 4.66, 95% CI: 3.05–7.12, p < .001), to wash hands (OR = 3.39, 95% CI: 1.81–6.35, p = < .001), to cancel personal/social activities (OR = 1.66, 95% CI: 1.22–2.27, p = .001), to avoid high-risk people (OR = 2.09, 95% CI: 1.42–3.08, p < .001), to avoid public places (OR = 1.73, 95% CI: 1.18–2.53, p = .005), and to avoid eating at a restaurant (OR = 2.07, 95% CI: 1.39–3.08, p < .001) (Table 2). Hispanics and Asians were also more likely than whites to take up all examined behaviors except washing hands and for Asians, avoiding high-risk people. Whites were least likely to take most preventive behaviors. On the other hand, the race/ethnicity was generally not significantly related to performing risky behaviors. Having more underlying chronic conditions appeared to be associated with wearing a mask and washing hands frequently, but not with any of the risky behaviors. Those with low education were less likely to wear a facemask, to cancel activities and to avoid public places but more likely to have close contact with non-household people and have visitors in their residence. Having a job was related to a greater likelihood of wearing a mask (OR = 1.50, 95% CI: 1.17–1.93, p = .001), washing hands (OR = 1.51, 95% CI: 1.06–2.16, p = .022), and contacting non-household people (OR = 2.49, 95% CI: 1.94–3.18, p < .001), but a smaller likelihood of avoiding high-risk people (OR = 0.80, 95% CI: 0.64–0.99, p = .044) or public places (OR = 0.76, 95% CI: 0.60–0.95, p = .018) and eating at a restaurant (OR = 0.73, 95% CI: 0.59–0.91, p = .005). People who lived in the states with a greater number of cases of infection were more likely to adopt preventive behaviors but did bit differ in risky behaviors. Those who trusted CNN more than Fox News were relatively more likely (48%) than those who trusted both equally or who did not trust either to wear a facemask (95% CI: 1.19–1.83, p = < .001), 63% more likely relatively to wash hands (95% CI: 1.15–2.30, p = .006), 28% more to cancel social/personal activities (95% CI: 1.09–1.50, p = 003), 63% more to avoid public places (95% CI: 1.32–2.02, p < .001), 51% more to avoid restaurants (95% CI: 1.24–1.84, p = < .001), 21% less to have close contact with non-household people (95% CI: 0.63–0.99, p = .045), 38% less to go to friend’s house (95% CI: 0.47–0.81, p < .001), 54% less to attend gatherings (95% CI: 0.26–0.82, p = .008), and 36% less to have visitors in residence (95% CI: 0.48–0.82, p = .001). Trusting Fox News more was not related to taking these behaviors. Those who trusted Fox News more were relatively more likely to have visitors in their residence (OR = 1.43, 95% CI: 1.06–1.93, p = .020); an effect in the opposite direction of that observed for those who trusted CNN more. Having a higher perceived risk of infection and dying was related to a greater likelihood of taking preventive behaviors.

## Discussion

Our study shows that the behavioral responses to COVID-19 differed by age and that over time the changes by age depended on the type of behaviors. When the pandemic began in March, older people did not differ from younger ones in taking preventive personal behaviors. As the pandemic progressed, older people quickly engaged in preventive personal behaviors to mitigate the COVID-19 infection. This supports our hypothesis that older people would be more careful and comply with recommended health practices. Our findings expand on previous knowledge by showing that older people became more likely to engage in personal healthy behaviors in the COVID-19 pandemic situation. Older persons may have become aware that they are more vulnerable to poor outcomes from the virus, and seen a greater need to follow better hygiene, quarantine and social distancing related behaviors. This awareness may have been absorbed quickly after the pandemic started and increases in infections and deaths were reported daily so that a significant part of the behavioral response observed between waves 1 and 4, had already occurred by wave 2.

At the same line, we found that older people were less likely to engage in some risky social behaviors than younger people a month after the pandemic started and this age difference continued. However, both younger and older people tended to resume potentially risky social behaviors as the pandemic progressed. This differential trend in personal behaviors such as mask wearing from social behaviors such as visiting with friends and family points out how people’s responses to the pandemic are quite mixed.

Our findings on increased risky behaviors over time among both older and younger people may be explained by the time reference and the type of behaviors. We compared risky behaviors only at wave 2 and wave 4 while we compared waves 1, 2 and 4 for preventive behaviors. Preventive behaviors, such as wearing a mask, avoiding public places and eating at restaurants were adopted quickly in the first month, between wave 1 and wave 2. For risky behaviors, we do not know what behaviors people engaged in at wave 1. It is possible that older people quickly stopped taking some risky behaviors, e.g., having contact with non-household members and going to another person’s house. However, after another month everyone, both younger and older, increased their risky behaviors perhaps because not engaging in social behaviors for an extended period of time may be difficult at all ages. Older people may continue their preventive personal behaviors; while they started loosening their rules for “risky behaviors.” People may stop risky social behaviors for a short period of time, but it may be difficult to continue for a longer time. As the pandemic is prolonged, people appear to loosen their restriction on interacting with family and friends. Some risky behaviors such as visiting or being visited by non-household friends and relatives may be behaviors that people cannot forgo for months no matter what their age is.

We also found that other characteristics are related to behavior during the ongoing pandemic. Being female, Black, Hispanic or Asian, having a higher education, having underlying conditions, residing in a state where the COVID-19 outbreak was more prevalent, trusting CNN more were linked to more preventive behaviors in response to COVID-19. This is consistent with previous studies that showed higher rates of healthy behaviors or behavior change among females [6], those with chronic conditions [8,12] and those with high socioeconomic status [19], but our results add to the list of factors affecting behavioral responses. Our study, however, differs from previous studies in that it is not based on a small or local sample, but on a relatively large sample selected and weighted to represent the behavior of all American adults in response to COVID-19. The consistently higher preventive behavioral response of Blacks, Hispanics, and Asians may reflect the knowledge that the pandemic was differentially affecting communities of color.

On the other hand, it is not clear why there was no association between being Black or Hispanic and engaging in risky behaviors. Many of the preventive personal activities examined may be required at work sites, the higher percent of minority members working outside home compared to whites may be related to racial/ethnic differences in taking these behaviors. On the other hand, the risky behaviors examined may be related to individual choice. Whites, Blacks and Hispanics may not differ in their desire to meet friends and family even though risky.

Our results show that those who are working and those with lower socioeconomic status may have more difficulty practicing recommended behaviors and be required to engage in more risky behaviors. Those who work out of the home may be more aware of the importance of wearing a mask or washing hands and be mandated to do so. However, their work may require them to be in contact with high-risk people, go to public places, and have close contact with non-household people which would increase their exposure to the virus. Proper work accommodations and protective guideline may need to be made for persons who are engaging in risky behaviors out of necessity. Our findings that those with low education wore a mask less, cancelled personal/social activities less, and had visitors in their residence more suggests that improving knowledge on the value of interventions may be targeted to those of lower education.

The number of COVID-19 cases in the state of residence was significantly related to people’s adoption of wearing a mask. While people may voluntarily wear a mask to protect themselves from the virus, they may also respond to state and local government mandates and campaigns on the importance of face covering in states where infection cases are greater. Some voluntary adoption of precautionary behaviors may also be based on people’s perception of their individual risk for infection or dying. Since our findings show that those who perceive their risk to be higher tend to engage in more preventive behaviors and fewer risky behaviors, additional accurate information and education may be critical in encouraging appropriate actions.

There is an association between trusted media sources and the likelihood of engaging in mitigating behaviors. We assume this is related to political attitudes; those who support the current federal government’s attitude, reaction and policies are less likely to reduce risky behaviors and adopt preventive behaviors. Our result shows the importance of media as well as political inclination on people’s response to the pandemic. Apolitical, scientifically based recommendations for behavior through the media could have changed behaviors.

It is possible that there are differential associations by age with behavioral factors other than age. For this reason, we compared model fit between models with associations estimated as the same across age groups and models with interactions between all variables and age groups. We found that the inclusion of the interaction terms modestly improved our model fit for some behaviors such as wearing a mask, avoiding high-risk people, avoiding public places, and avoiding restaurants, but not for others. Factors that have a significant interaction with age include gender, having a job, education, state case numbers, political orientation and assessed risk of infection. These factors improve the model fit compared to our original models; however, they didn’t change the observed association of age with behaviors.

There are some limitations we need to acknowledge. First, we are aware that our comparison between waves 2 and 4 may not include part of the behavioral modifications made right after the pandemic started. March (wave 1) was just the beginning of the pandemic when people might have been unaware of what behavioral modifications were important as infections and deaths were not yet prevalent. April (wave 2) was a month after the pandemic started and the impact of COVID-19 on people’s lives was becoming clearer. Some behavioral changes may have already occurred between waves 1 and 2. Second, our measure of the number of infections by state may not capture the diversity of infection within states which may differ by urbanicity and other factors. Third, while the UAS questions about behavioral modifications were developed to control the tendency of survey participants to provide more socially desirable responses by asking people to consider only actions they had personally taken. Social desirability might have affected people’s responses about adopting preventive behaviors and the effect could differ by age and over time, particularly when recommendations changed across time and differed across states leading to a lack of clarity as to expected behaviors. Fourth, while very few people resided in residential facilities, a few are included in the survey, and they cannot be distinguished in the data.

It is encouraging to observe older people taking more preventive personal behaviors as the pandemic progressed, which may have alleviated their risk of infection. However, at the same time, it is concerning that people started loosening observance of recommendations to avoid risky behaviors, particularly older people who could have more adverse consequences from meeting with family and friends. Because there is no immediate cure and little treatment for the condition, while scientists are attempting to develop and distribute a vaccine, proper personal and social practices may be the only route to reducing infection for older people. Given more severe consequences for older people once infected, older people should be strongly encouraged to continue taking preventive personal behaviors and not to increase risky behaviors since the virus could be transmitted during these activities.

## Supporting information

S1 Fig Predicted probabilities of performing preventive personal behaviors in response to COVID-19 by age: Wave 1 (03/10/20-03/31/20), wave 2 (04/01/20-04/28/20) and wave 4 (04/29/20-05/26/20). (PDF)

S2 Fig Predicted probabilities of performing risky social behaviors in response to COVID-19 by age: Wave 2 (04/01/20-04/28/20) and wave 4 (04/29/20-05/26/20). (PDF)

S1 Table Odds ratios for age and other factors related to preventive personal behaviors: Wave 1 (03/10/20-03/31/20), wave 2 (04/01/20-04/28/20) and wave 4 (04/29/20-05/26/20). (DOCX)

S2 Table Odds ratios for age and other factors related to risky social behaviors: Wave 2 (04/01/20-04/28/20) and wave 4 (04/29/20-05/26/20). (DOCX)
