# Supplementary material for: How does age affect personal and social reactions to COVID-19: Results from the national Understanding America Study
Source: PLoS One. 2020 Nov 10;15(11):e0241950. doi: 10.1371/journal.pone.0241950 (PMC7654776; doi:10.1371/journal.pone.0241950)
Supplement: S2 Fig — (PDF) [file pone.0241950.s002.pdf]

S2 Fig. Predicted Probabilities of Performing Risky Social Behaviors in Response to COVID-19 by Age: Wave 2 (04/01/20-04/28/20) and Wave 4 (04/29/20-05/26/20)

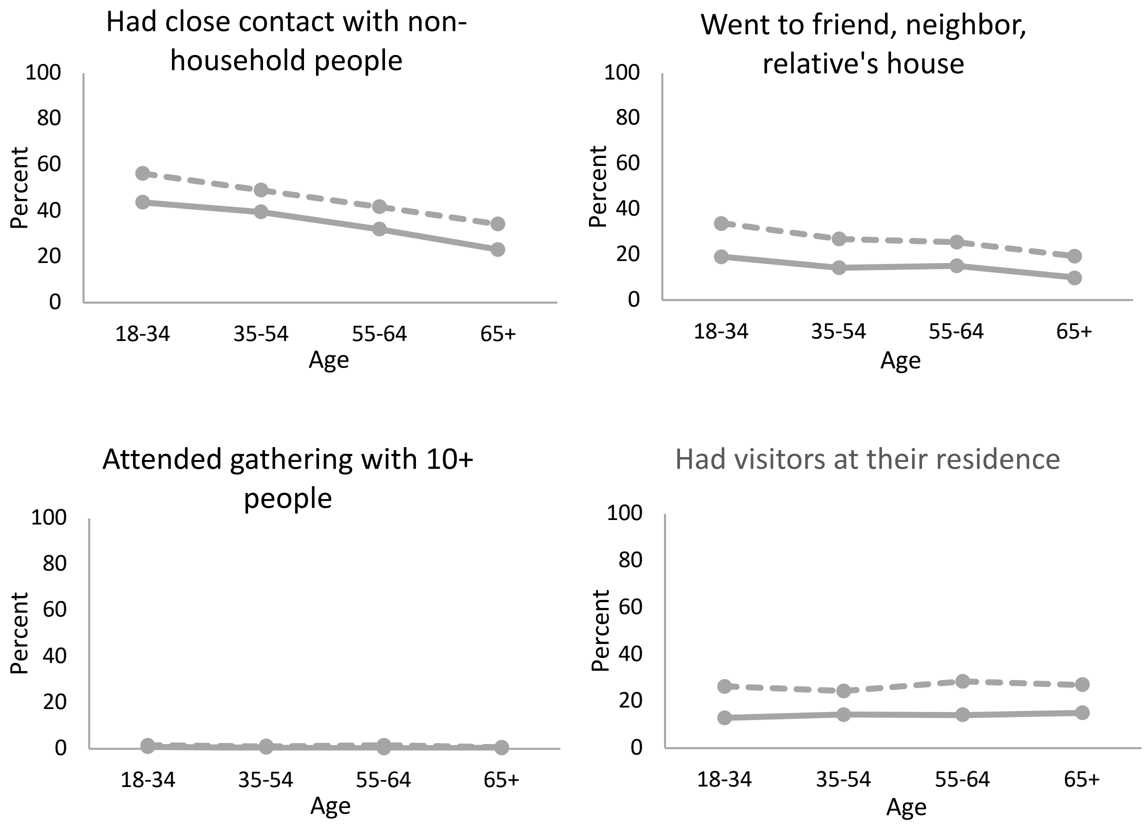

Predicted probabilities were calculated based on Table 3.
