# Supplementary material for: How does age affect personal and social reactions to COVID-19: Results from the national Understanding America Study
Source: PLoS One. 2020 Nov 10;15(11):e0241950. doi: 10.1371/journal.pone.0241950 (PMC7654776; doi:10.1371/journal.pone.0241950)
Supplement: S1 Table — (DOCX) [file pone.0241950.s003.docx]

S1 Table. Odds Ratios for Age and Other Factors Related to Preventive Personal Behaviors: Wave 1 (03/10/20-03/31/20), Wave 2 (04/01/20-04/28/20) and Wave 4 (04/29/20-05/26/20)

|  | **Wore mask** | | | | **Washed hands** | | | | **Canceled personal/ social activities** | | | | **Avoided high-risk people** | | | | **Avoided public places** | | | | **Avoided restaurant** | | | |
| --- | --- | --- | --- | --- | --- | --- | --- | --- | --- | --- | --- | --- | --- | --- | --- | --- | --- | --- | --- | --- | --- | --- | --- | --- |
|  | OR | 95% CI | | p | OR | 95% CI | | p | OR | 95% CI | | p | OR | 95% CI | | p | OR | 95% CI | | p | OR | 95% CI | | p |
| Age (18-34 years as reference) |  |  |  |  |  |  |  |  |  |  |  |  |  |  |  |  |  |  |  |  |  |  |  |  |
| 35-54 | 0.98 | 0.54 | 1.78 | 0.946 | 0.98 | 0.57 | 1.68 | 0.950 | 0.97 | 0.70 | 1.35 | 0.859 | 1.04 | 0.74 | 1.47 | 0.813 | 1.16 | 0.82 | 1.66 | 0.405 | 1.18 | 0.81 | 1.71 | 0.385 |
| 55-64 | 0.73 | 0.38 | 1.40 | 0.344 | 1.27 | 0.68 | 2.37 | 0.449 | 0.73 | 0.51 | 1.06 | 0.103 | 0.98 | 0.67 | 1.44 | 0.917 | 0.96 | 0.65 | 1.43 | 0.850 | 1.02 | 0.68 | 1.54 | 0.917 |
| 65+ | 0.73 | 0.36 | 1.46 | 0.370 | 1.45 | 0.77 | 2.72 | 0.247 | 0.90 | 0.62 | 1.30 | 0.577 | 0.95 | 0.64 | 1.42 | 0.813 | 1.06 | 0.69 | 1.61 | 0.798 | 0.80 | 0.53 | 1.22 | 0.301 |
| Wave (wave 1 as reference) |  |  |  |  |  |  |  |  |  |  |  |  |  |  |  |  |  |  |  |  |  |  |  |  |
| Wave 2 | 28.33 | 15.31 | 52.42 | <.001 | 4.14 | 1.93 | 8.87 | <.001 | 10.57 | 7.05 | 15.85 | <.001 | 15.37 | 9.51 | 24.84 | <.001 | 19.25 | 11.45 | 32.37 | <.001 | 29.77 | 17.66 | 50.18 | <.001 |
| Wave 4 | 410.96 | 183.32 | 921.26 | <.001 | 1.03 | 0.55 | 1.93 | 0.929 | 2.29 | 1.61 | 3.26 | <.001 | 6.42 | 4.01 | 10.27 | <.001 | 7.68 | 4.77 | 12.36 | <.001 | 12.35 | 7.82 | 19.49 | <.001 |
| Age*wave interaction |  |  |  |  |  |  |  |  |  |  |  |  |  |  |  |  |  |  |  |  |  |  |  |  |
| Age 35-54*wave 2 | 1.50 | 0.76 | 2.98 | 0.244 | 1.29 | 0.52 | 3.21 | 0.588 | 1.13 | 0.70 | 1.84 | 0.613 | 1.02 | 0.58 | 1.80 | 0.951 | 1.17 | 0.64 | 2.14 | 0.616 | 1.18 | 0.65 | 2.14 | 0.590 |
| Age 35-54*wave 4 | 1.21 | 0.54 | 2.72 | 0.646 | 2.74 | 1.28 | 5.86 | 0.009 | 1.14 | 0.74 | 1.76 | 0.541 | 1.22 | 0.72 | 2.08 | 0.466 | 1.34 | 0.78 | 2.32 | 0.292 | 1.44 | 0.86 | 2.40 | 0.165 |
| Age 55-64*wave 2 | 3.16 | 1.55 | 6.45 | 0.002 | 2.66 | 0.95 | 7.49 | 0.064 | 1.56 | 0.91 | 2.65 | 0.105 | 1.23 | 0.65 | 2.34 | 0.519 | 3.00 | 1.52 | 5.94 | 0.002 | 3.19 | 1.67 | 6.11 | <.001 |
| Age 55-64*wave 4 | 3.64 | 1.59 | 8.30 | 0.002 | 4.73 | 1.95 | 11.50 | 0.001 | 2.08 | 1.30 | 3.33 | 0.002 | 1.44 | 0.79 | 2.62 | 0.229 | 2.96 | 1.62 | 5.41 | <.001 | 3.01 | 1.73 | 5.25 | <.001 |
| Age 65+*wave 2 | 3.05 | 1.42 | 6.52 | 0.004 | 1.20 | 0.44 | 3.25 | 0.726 | 1.47 | 0.89 | 2.43 | 0.133 | 1.35 | 0.72 | 2.54 | 0.346 | 3.37 | 1.68 | 6.74 | 0.001 | 7.23 | 3.61 | 14.47 | <.001 |
| Age 65+*wave 4 | 3.94 | 1.68 | 9.24 | 0.002 | 3.90 | 1.65 | 9.22 | 0.002 | 1.66 | 1.05 | 2.60 | 0.028 | 1.98 | 1.11 | 3.54 | 0.021 | 2.90 | 1.56 | 5.40 | 0.001 | 6.85 | 3.86 | 12.18 | <.001 |
| Female | 1.42 | 1.19 | 1.70 | <.001 | 2.61 | 1.97 | 3.46 | <.001 | 1.30 | 1.13 | 1.49 | <.001 | 1.30 | 1.10 | 1.54 | 0.002 | 1.31 | 1.10 | 1.56 | 0.003 | 1.26 | 1.07 | 1.48 | 0.005 |
| Living alone | 0.91 | 0.71 | 1.17 | 0.451 | 0.65 | 0.46 | 0.93 | 0.018 | 0.81 | 0.66 | 0.98 | 0.029 | 0.89 | 0.71 | 1.11 | 0.308 | 0.77 | 0.60 | 0.98 | 0.037 | 0.87 | 0.68 | 1.10 | 0.239 |
| Race/ethnicity (white as reference) |  |  |  |  |  |  |  |  |  |  |  |  |  |  |  |  |  |  |  |  |  |  |  |  |
| Black | 4.66 | 3.05 | 7.12 | <.001 | 3.39 | 1.81 | 6.35 | <.001 | 1.66 | 1.22 | 2.27 | 0.001 | 2.09 | 1.42 | 3.08 | <.001 | 1.73 | 1.18 | 2.53 | 0.005 | 2.07 | 1.39 | 3.08 | <.001 |
| Hispanic | 2.12 | 1.48 | 3.03 | <.001 | 3.59 | 2.14 | 6.02 | <.001 | 2.20 | 1.68 | 2.88 | <.001 | 1.70 | 1.20 | 2.42 | 0.003 | 2.13 | 1.48 | 3.06 | <.001 | 1.83 | 1.31 | 2.56 | <.001 |
| Asian | 3.26 | 1.85 | 5.73 | <.001 | 1.58 | 0.69 | 3.59 | 0.278 | 1.94 | 1.30 | 2.89 | 0.001 | 1.38 | 0.83 | 2.31 | 0.219 | 3.03 | 1.76 | 5.22 | <.001 | 3.40 | 2.07 | 5.59 | <.001 |
| Other | 1.73 | 1.14 | 2.65 | 0.011 | 1.54 | 0.80 | 2.96 | 0.194 | 1.12 | 0.79 | 1.59 | 0.525 | 1.39 | 0.86 | 2.24 | 0.176 | 1.09 | 0.68 | 1.73 | 0.725 | 1.15 | 0.72 | 1.81 | 0.561 |
| Education (16+ years as reference) |  |  |  |  |  |  |  |  |  |  |  |  |  |  |  |  |  |  |  |  |  |  |  |  |
| 13-15 years | 0.92 | 0.70 | 1.20 | 0.542 | 1.04 | 0.68 | 1.61 | 0.844 | 1.09 | 0.88 | 1.34 | 0.431 | 0.96 | 0.74 | 1.24 | 0.767 | 1.21 | 0.91 | 1.62 | 0.189 | 1.14 | 0.89 | 1.46 | 0.311 |
| 12 years | 1.02 | 0.78 | 1.35 | 0.867 | 0.88 | 0.57 | 1.34 | 0.545 | 0.77 | 0.62 | 0.94 | 0.011 | 0.85 | 0.66 | 1.10 | 0.211 | 0.70 | 0.54 | 0.92 | 0.010 | 0.86 | 0.67 | 1.11 | 0.252 |
| 0-11 years | 0.65 | 0.47 | 0.89 | 0.008 | 0.74 | 0.46 | 1.20 | 0.219 | 0.64 | 0.51 | 0.81 | 0.000 | 0.95 | 0.71 | 1.27 | 0.747 | 0.72 | 0.53 | 0.98 | 0.039 | 0.87 | 0.65 | 1.17 | 0.359 |
| Number of chronic conditions | 1.12 | 1.03 | 1.21 | 0.005 | 1.14 | 1.02 | 1.27 | 0.026 | 0.96 | 0.91 | 1.02 | 0.185 | 1.00 | 0.94 | 1.08 | 0.930 | 1.04 | 0.96 | 1.12 | 0.323 | 0.99 | 0.92 | 1.07 | 0.828 |
| Currently having a job | 1.50 | 1.17 | 1.93 | 0.001 | 1.51 | 1.06 | 2.16 | 0.022 | 0.93 | 0.78 | 1.11 | 0.436 | 0.80 | 0.64 | 0.99 | 0.044 | 0.76 | 0.60 | 0.95 | 0.018 | 0.73 | 0.59 | 0.91 | 0.005 |
| In poverty | 1.07 | 0.76 | 1.52 | 0.687 | 0.65 | 0.43 | 1.01 | 0.053 | 0.89 | 0.69 | 1.15 | 0.370 | 0.88 | 0.65 | 1.18 | 0.400 | 0.87 | 0.63 | 1.22 | 0.425 | 0.91 | 0.66 | 1.25 | 0.570 |
| Number of COVID-19 cases in the state of residence (per 1,000) | 1.01 | 1.00 | 1.02 | 0.004 | 1.00 | 1.00 | 1.01 | 0.397 | 1.00 | 1.00 | 1.00 | 0.836 | 1.00 | 1.00 | 1.00 | 0.677 | 1.00 | 0.99 | 1.00 | 0.477 | 1.00 | 1.00 | 1.01 | 0.054 |
| Political inclination (equal trust or no trust as reference) |  |  |  |  |  |  |  |  |  |  |  |  |  |  |  |  |  |  |  |  |  |  |  |  |
| Trust Fox news  more | 0.87 | 0.67 | 1.12 | 0.278 | 1.08 | 0.75 | 1.54 | 0.690 | 0.92 | 0.77 | 1.11 | 0.408 | 0.87 | 0.69 | 1.09 | 0.236 | 0.91 | 0.71 | 1.15 | 0.418 | 0.72 | 0.57 | 0.91 | 0.006 |
| Trust CNN more | 1.48 | 1.19 | 1.83 | <.001 | 1.63 | 1.15 | 2.30 | 0.006 | 1.28 | 1.09 | 1.50 | 0.003 | 1.21 | 0.99 | 1.47 | 0.063 | 1.63 | 1.32 | 2.02 | <.001 | 1.51 | 1.24 | 1.84 | <.001 |
| Perceived risk for infection | 1.01 | 1.00 | 1.01 | 0.001 | 1.00 | 0.99 | 1.00 | 0.423 | 1.01 | 1.00 | 1.01 | 0.001 | 1.01 | 1.00 | 1.01 | 0.016 | 1.01 | 1.00 | 1.01 | 0.007 | 1.01 | 1.00 | 1.01 | 0.008 |
| Perceived risk for dying | 1.00 | 1.00 | 1.01 | 0.097 | 1.01 | 1.01 | 1.02 | <.001 | 1.01 | 1.01 | 1.01 | <.001 | 1.01 | 1.00 | 1.01 | <.001 | 1.01 | 1.01 | 1.02 | <.001 | 1.01 | 1.00 | 1.01 | 0.001 |
| N | 4,825 | | | | 4,825 | | | | 4,805 | | | | 4,690 | | | | 4,812 | | | | 4,812 | | | |
| Wald χ2 | 839.43 | | | | 266.48 | | | | 886.36 | | | | 683.70 | | | | 785.00 | | | | 931.79 | | | |
| Prob > χ2 | 0.0000 | | | | 0.0000 | | | | 0.0000 | | | | 0.0000 | | | | 0.0000 | | | | 0.0000 | | | |
| Log pseudolikelihood | -5544.50 | | | | -2803.69 | | | | -7711.72 | | | | -5971.95 | | | | -5581.58 | | | | -5979.43 | | | |
