# Supplementary material for: How does age affect personal and social reactions to COVID-19: Results from the national Understanding America Study
Source: PLoS One. 2020 Nov 10;15(11):e0241950. doi: 10.1371/journal.pone.0241950 (PMC7654776; doi:10.1371/journal.pone.0241950)
Supplement: S2 Table — (DOCX) [file pone.0241950.s004.docx]

S2 Table. Odds Ratios for Age and Other Factors Related to Risky Social Behaviors: Wave 2 (04/01/20-04/28/20) and Wave 4 (04/29/20-05/26/20)

|  | **Had close contact with non-household people** | | | | **Went to friend, relative’s residence** | | | | | **Attended gathering of 10+ people** | | | | **Had visitors in residence** | | | |
| --- | --- | --- | --- | --- | --- | --- | --- | --- | --- | --- | --- | --- | --- | --- | --- | --- | --- |
|  | OR | 95% CI | | p | OR | 95% CI | | p | | OR | 95% CI | | p | OR | 95% CI | | p |
| Age (18-34 years as reference) |  |  |  |  |  |  |  | |  |  |  |  |  |  |  |  |  |
| 35-54 | 0.74 | 0.51 | 1.09 | 0.126 | 0.58 | 0.37 | 0.91 | | 0.019 | 0.68 | 0.23 | 2.04 | 0.488 | 1.19 | 0.73 | 1.92 | 0.487 |
| 55-64 | 0.56 | 0.36 | 0.87 | 0.010 | 0.66 | 0.39 | 1.11 | | 0.114 | 0.53 | 0.15 | 1.85 | 0.320 | 1.12 | 0.66 | 1.91 | 0.669 |
| 65+ | 0.44 | 0.28 | 0.69 | <.001 | 0.37 | 0.22 | 0.63 | | <.001 | 0.73 | 0.21 | 2.55 | 0.624 | 1.26 | 0.73 | 2.19 | 0.408 |
| Wave (wave 2 as reference) |  |  |  |  |  |  |  | |  |  |  |  |  |  |  |  |  |
| Wave 4 | 1.50 | 1.22 | 1.83 | <.001 | 1.80 | 1.43 | 2.26 | | <.001 | 1.47 | 0.84 | 2.58 | 0.174 | 1.88 | 1.45 | 2.44 | <.001 |
| Age*wave interaction |  |  |  |  |  |  |  | |  |  |  |  |  |  |  |  |  |
| Age 35-54*wave 4 | 0.78 | 0.48 | 1.27 | 0.319 | 0.96 | 0.56 | 1.65 | | 0.883 | 1.29 | 0.32 | 5.27 | 0.720 | 0.64 | 0.35 | 1.16 | 0.140 |
| Age 55-64*wave 4 | 0.77 | 0.45 | 1.30 | 0.327 | 0.76 | 0.42 | 1.38 | | 0.370 | 3.08 | 0.69 | 13.83 | 0.141 | 0.91 | 0.48 | 1.72 | 0.769 |
| Age 65+*wave 4 | 0.94 | 0.56 | 1.55 | 0.799 | 0.89 | 0.49 | 1.61 | | 0.705 | 1.06 | 0.22 | 5.19 | 0.943 | 0.74 | 0.40 | 1.36 | 0.330 |
| Female | 0.80 | 0.66 | 0.97 | 0.021 | 0.93 | 0.75 | 1.16 | | 0.539 | 0.92 | 0.61 | 1.38 | 0.674 | 0.96 | 0.77 | 1.19 | 0.724 |
| Living alone | 1.54 | 1.20 | 1.98 | 0.001 | 1.87 | 1.38 | 2.54 | | <.001 | 1.41 | 0.82 | 2.44 | 0.215 | 1.05 | 0.76 | 1.45 | 0.756 |
| Race/ethnicity (white as reference) |  |  |  |  |  |  |  | |  |  |  |  |  |  |  |  |  |
| Black | 0.99 | 0.65 | 1.52 | 0.962 | 0.82 | 0.51 | 1.33 | | 0.420 | 1.23 | 0.54 | 2.79 | 0.620 | 0.87 | 0.54 | 1.40 | 0.557 |
| Hispanic | 1.10 | 0.76 | 1.59 | 0.630 | 0.72 | 0.47 | 1.13 | | 0.154 | 1.58 | 0.78 | 3.18 | 0.200 | 0.99 | 0.64 | 1.53 | 0.955 |
| Asian | 0.71 | 0.41 | 1.23 | 0.221 | 0.68 | 0.36 | 1.27 | | 0.222 | 1.22 | 0.34 | 4.45 | 0.759 | 0.51 | 0.26 | 0.99 | 0.046 |
| Other | 1.78 | 1.12 | 2.83 | 0.016 | 1.40 | 0.81 | 2.44 | | 0.230 | 2.43 | 0.91 | 6.50 | 0.077 | 1.70 | 1.04 | 2.79 | 0.035 |
| Education (16+ years as reference) |  |  |  |  |  |  |  | |  |  |  |  |  |  |  |  |  |
| 13-15 years | 1.28 | 0.96 | 1.71 | 0.098 | 1.09 | 0.77 | 1.55 | | 0.610 | 0.65 | 0.32 | 1.31 | 0.226 | 0.99 | 0.69 | 1.40 | 0.944 |
| 12 years | 1.97 | 1.47 | 2.65 | <.001 | 1.27 | 0.90 | 1.79 | | 0.169 | 0.71 | 0.36 | 1.40 | 0.325 | 1.44 | 1.02 | 2.04 | 0.039 |
| 0-11 years | 1.49 | 1.06 | 2.08 | 0.021 | 1.16 | 0.79 | 1.71 | | 0.456 | 0.57 | 0.28 | 1.17 | 0.123 | 1.54 | 1.05 | 2.26 | 0.028 |
| Number of chronic conditions | 1.01 | 0.93 | 1.09 | 0.811 | 0.97 | 0.88 | 1.06 | | 0.486 | 0.91 | 0.75 | 1.10 | 0.316 | 1.09 | 0.99 | 1.19 | 0.067 |
| Currently having a job | 2.49 | 1.94 | 3.18 | <.001 | 1.30 | 0.99 | 1.72 | | 0.061 | 1.35 | 0.77 | 2.39 | 0.296 | 1.23 | 0.93 | 1.62 | 0.151 |
| In poverty | 0.69 | 0.50 | 0.96 | 0.027 | 0.81 | 0.55 | 1.20 | | 0.297 | 1.44 | 0.73 | 2.83 | 0.291 | 1.01 | 0.67 | 1.53 | 0.944 |
| Number of COVID-19 cases in the state of residence (per 1,000) | 1.00 | 0.99 | 1.00 | 0.236 | 1.00 | 0.99 | 1.00 | | 0.379 | 1.00 | 0.98 | 1.01 | 0.579 | 1.00 | 1.00 | 1.01 | 0.657 |
| Political inclination (equal trust or no trust as reference) |  |  |  |  |  |  |  | |  |  |  |  |  |  |  |  |  |
| Trust Fox news  more | 1.11 | 0.86 | 1.44 | 0.419 | 1.19 | 0.88 | 1.61 | | 0.249 | 1.49 | 0.87 | 2.55 | 0.145 | 1.43 | 1.06 | 1.93 | 0.020 |
| Trust CNN more | 0.79 | 0.63 | 0.99 | 0.045 | 0.62 | 0.47 | 0.81 | | <.001 | 0.46 | 0.26 | 0.82 | 0.008 | 0.62 | 0.48 | 0.82 | 0.001 |
| Perceived risk for infection | 0.99 | 0.99 | 1.00 | <.001 | 1.00 | 0.99 | 1.00 | | 0.113 | 0.99 | 0.98 | 1.00 | 0.150 | 1.00 | 0.99 | 1.00 | 0.075 |
| Perceived risk for dying | 1.01 | 1.00 | 1.01 | 0.006 | 1.00 | 0.99 | 1.00 | | 0.772 | 1.00 | 0.99 | 1.01 | 0.873 | 1.00 | 0.99 | 1.01 | 0.813 |
| N | 4,759 | | | | 4,820 | | | | | 4,819 | | | | 4,815 | | | |
| Wald χ2 | 298.04 | | | | 265.40 | | | | | 105.48 | | | | 217.25 | | | |
| Prob > χ2 | 0.0000 | | | | 0.0000 | | | | | 0.0000 | | | | 0.0000 | | | |
| Log pseudolikelihood | -5189.99 | | | | -4386.59 | | | | | -915.73 | | | | -4451.71 | | | |
